# Identification of a novel pathogenic variant in the *MYH3* gene in a five‐generation family with CPSFS1A (Contractures, Pterygia, and Spondylocarpotarsal Fusion Syndrome 1A)

**DOI:** 10.1002/mgg3.1440

**Published:** 2020-08-07

**Authors:** Jing Zhang, Wen‐Qi Chen, Si‐Wen Wang, Shao‐Xiong Wang, Mei Yu, Qing Guo, Ya‐Dong Yu

**Affiliations:** ^1^ Center of Prenatal Diagnosis Shijiazhuang Obstetrics and Gynecology Hospital Shijiazhuang China; ^2^ Department of Hand Surgery The Third Hospital of Hebei Medical University Shijiazhuang China

**Keywords:** CPSFS1A, distal arthrogryposis, *MYH3*, whole‐exome sequencing

## Abstract

**Background:**

Distal arthrogryposis (DA) is a group of rare Mendelian conditions that demonstrate heterogeneity with respect to genetics and phenotypes. Ten types of DAs, which collectively involve six genes, have been reported. Among them, the *MYH3* gene causes several types of arthrogryposis conditions and therefore has a pivotal role in the skeletal and muscle development of the fetus. For this study, we recruited a five‐generation Chinese family with members presenting DA features and phenotypic variability. Further clinical study characterized it as CPSFS1A (Contractures, Pterygia, and Spondylocarpotarsal Fusion Syndrome 1A).

**Methods:**

Genomic DNA was extracted from eight family members, including one fetus. Whole‐exome sequencing (WES) was then conducted on the proband's sample, followed by Sanger sequencing as validation for each of the participants. In silico analysis was performed. Western blotting (WB) detection and pathological staining were conducted on skeletal muscle tissue of the induced fetus after prenatal diagnosis.

**Results:**

A novel heterozygous pathogenic variant, namely NM_002470.3: c.3044_3047delinsTCAATTTGTT: p.E1015_D1016delinsVNLF in the *MYH3* gene, was identified and shown to be cosegregated with the condition in the subject family. This variant resulted in the replacement of amino‐acid residues E1015 and D1016 by a string of VNLFs. The pregnancy was selectively terminated because the fetus was genetically affected. However, the WB and pathological results did not indicate a significant change in the norm.

**Conclusions:**

Our study expanded the variant spectrum of CPSFS1A, in addition to which it provided solid evidence for the appropriateness of genetic counseling and pregnancy management for the family. The results may also provide further insight into the molecular mechanism of *MYH3*.

## Graphical abstract

We have reported for the first time in a Chinese five‐generation family, a novel pathogenic variant c.3044_3047delinsTCAATTTGTT in the MYH3 gene causing CPSFS1A.

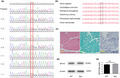

## 1 INTRODUCTION

Distal arthrogryposis (DA) syndromes comprise a group of non‐progressive muscle diseases mainly characterized by multiple congenital limb contractures (Hall, Reed, & Greene, 1982). The commonly accepted clinical definition of these inherited limb malformation disorders was congenital contractures in at least two identical body parts without constitutional neurological and/or muscular disorder affecting limb function (Bamshad, Jorde, & Carey, 1996), and many patients displayed craniofacial anomalies (Beals, 2005). In 1996, the classification of DA was revised and several additional conditions were categorized (Bamshad et al., 1996), and the contractures, pterygia, and spondylocarpotarsal fusion syndrome 1A (CPSFS1A, formerly known as DA8, MIM #178110) was thus included due to its symptomatic similarity with other types of DA. To date, at least ten DA conditions with overlapping and distinct phenotypes have been reported, involving six genes, namely *TPM2* (MIM *190990), *TNNI2* (MIM *191043), *PIEZO2* (MIM *613629), *TNNT3* (MIM *600692), *ECEL1* (MIM *605896), and *MYH3* (MIM *160720), consistent with autosomal dominant (AD) and recessive (AR) genetic patterns, respectively (https://www.omim.org/, 2020/Mar/30).

CPSFS1A, as initially described by Kawira and Bender (Kawira & Bender, 1985), is caused by pathogenic variants in the *MYH3* gene (Chong et al., 2015), which encodes the myosin heavy‐chain isoform 3 (Yoon, Seiler, Kucherlapati, & Leinwand, 1992). Conforming to an AD pattern, it is characterized by multiple pterygia, congenital contractures of the limbs, scoliosis hemivertebrae, and vertebral fusion (Cameron‐Christie et al., 2018; Chong et al., 2015; Scala et al., 2018). However, CPSFS1A is widely variable with respect to phenotype (Kimber, Tajsharghi, Kroksmark, Oldfors, & Tulinius, 2012). Recently, a homogeneous AR condition, namely CPSFS1AB (MIM #618469), was described by Cameron‐Christie et al., being caused by compound heterozygous variants in *MYH3* (Cameron‐Christie et al., 2018). Severe congenital musculoskeletal abnormalities in such disorders endorse an important role for *MYH3* in primary embryonic skeletal and muscular development (Tajsharghi & Oldfors, 2013). Pathogenic variants in *MYH3* were associated with other types of DAs such as Freeman‐Sheldon syndrome (FSS, a.k.a. DA2A, MIM #193700) as well as Sheldon‐Hall syndrome (SHS, a.k.a. DA2B3, MIM #618436; Tajsharghi et al., 2008; Toydemir et al., 2006).

Next‐generation sequencing (NGS), particularly whole‐exome sequencing (WES), has been widely used in the diagnosis of fetal and neonatal malformations and prenatal diagnosis, particularly those in which the phenotypes are not distinguishable (Bae et al., 2016; Fu et al., 2018; Monaghan, Leach, Pekarek, Prasad, & Rose, 2020; Todd et al., 2015). In our previous study, the trio‐WES strategy has been demonstrated to be effective in elucidating the etiology of fetal skeletal dysplasia and in providing solid evidence for the genetic counseling of affected families (Yang, Shen, et al., 2019).

As part of this study, we investigated the genetic cause of an inherited CPSFS1A and conducted prenatal diagnosis for the suspected fetal involvement, doing so for the first time with respect to a five‐generation Chinese family. Additionally, the potential impact of a detected variant on protein function was discussed in the context of the in silico survey and review of the literature.

## 2 MATERIALS AND METHODS

### 2.1 Ethical compliance

This study was approved by the Research Ethics Committee of the Shijiazhuang Obstetrics and Gynecology Hospital (approval no. 20200043). Each of the participants signed an informed consent.

### 2.2 Subjects

**Figure 1:**
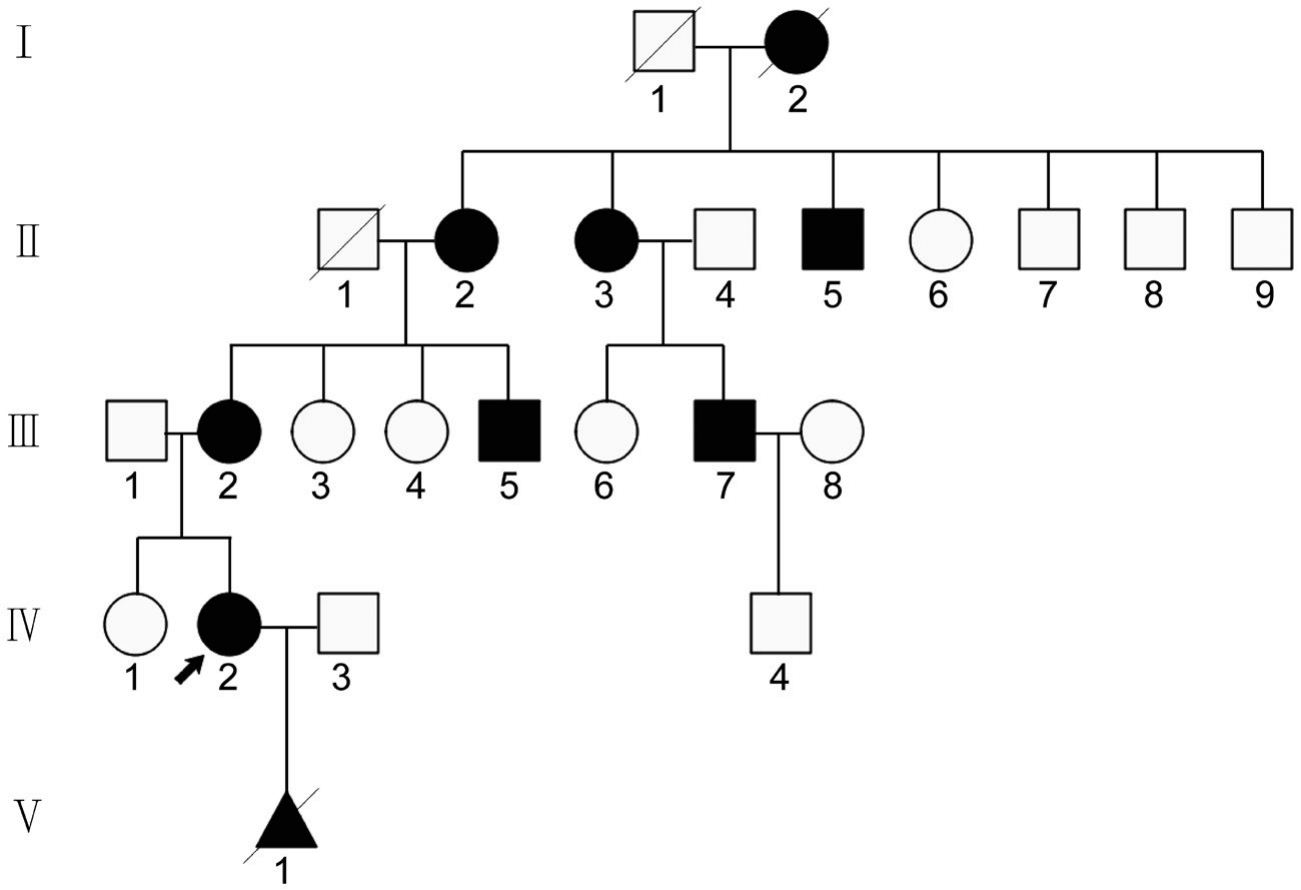
Pedigree of the family: The dark arrow indicates the proband. The dark shades represent members affected by the CPSFS1 condition

**Figure 2:**
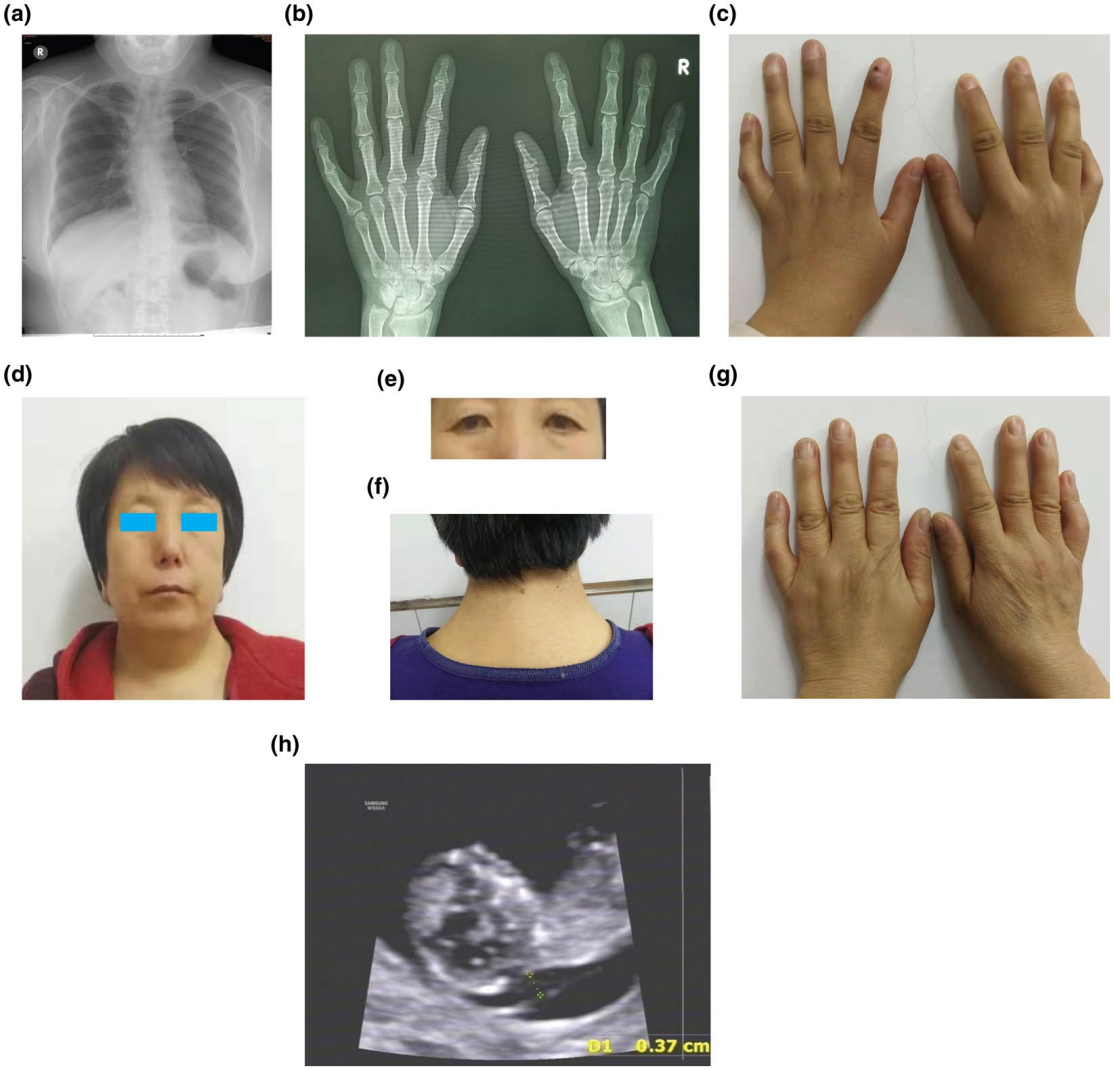
Clinical features and images of the subjects: (a–c) Images of x‐ray chest/hands and hand exterior of the proband. (d–g) Images of the facial features, eyes, neck pterygium, and hand exterior of Ⅲ:2. (h) Ultrasonoscopy of the fetus of the proband at 11^+4d^ gestational weeks

A five‐generation Chinese family with inherited DA, including one pregnant proband was referred to the Prenatal Diagnosis Center at the Shijiazhuang Obstetrics and Gynecology Hospital. An autosomal dominant genetic pattern was determined based on the family history survey (Figure 1). The proband and her mother both had distinct facial features, including down‐slanted palpebral fissures, ptosis of the eyelids, a long nasal bridge, a small mouth with downturned corners, neck webbing, and congenital limb contractures (Figure 2b–g). Meanwhile, the mental, intellectual, menstruation, and fertility development of them was normal. The x‐ray results indicated that the proband had severe scoliosis (Figure 2a) and carpal fusion and dysmorphia (Figure 2b). There were twenty‐four members in this family, and eight of them suffered from DA, while the other 15 were of normal phenotype. In total, 12 members (Ⅱ:2; Ⅱ:3; Ⅱ:5; Ⅲ:2; Ⅲ:3; Ⅲ:4; Ⅲ:5; Ⅲ:7; Ⅳ:1; Ⅳ:2; Ⅳ:3; V:1) were recruited (detailed information shown in Table 1) and eight were submitted to genetic testing (except for Ⅱ:3; Ⅱ:5; Ⅲ:4; Ⅲ:7).

**Table 1:** Clinical information of recruited subjects

Subject No.	Age (year)	Height (cm)	Congenital limb contractures	Neck pterygium	Congenital scoliosis
II:2	72	141	Third, fourth, and fifth fingers	(+)	(+)
II:3	70	139	Fifth finger	(+)	(+)
II:5	69	155	First and fifth fingers	(+)	(+)
III:2	51	140	Fifth finger	(+)	(+)
III:3	50	160	(−)	(−)	(−)
III:4	49	162	(−)	(−)	(−)
III:5	48	155	First, second, and fifth fingers	(+)	(+)
III:7	46	156	Second and fifth fingers	(+)	(+)
Ⅳ:1	29	163	(−)	(−)	(−)
Ⅳ:2 (P*)	28	143	Second and fifth fingers	(+)	(+)
Ⅳ:3	28	170	(−)	(−)	(−)
V:1 (F*)	/	/	/	/	/

F*, Fetus; P*, Proband.

### 2.3 Genomic DNA extraction

We collected 3 ml of peripheral blood from each of the eight members, doing so by means of BD Vacutainer™ tubes (BD Biosciences, San Jose, California, USA) for further analysis. Genomic DNA was extracted by means of QIAamp DNA Blood mini‐kits (Qiagen Sciences, Inc.) according to the manufacturer's protocol.

### 2.4 Whole‐exome sequencing

Enrichment of the target‐region sequences was performed by means of the Agilent Sure Select Human Exon Sequence Capture Kit. The sequencing libraries were quantified using the Illumina DNA Standards and Primer Premix Kit (Kapa Biosystems), massively parallel‐sequenced using the Illumina XTEN platform and then massively parallel‐sequenced again using the Illumina XTEN. After sequencing and filtering out the low‐quality readings, the high‐quality readings were compared to the human genome reference sequence [hg19]. The GATK software was used to identify variants (https://software.broadinstitute.org/gatk/). The suspected pathogenic variant was validated by Sanger sequencing using ABI 3730 Automated Sequencer (Applied Biosystems). The mutations were identified by sequence alignment with the NCBI Reference Sequence (NG 011537.1) using Chromas 2.33. Amino‐acid sequence conservation analysis of the MYH3 protein was performed using the NCBI BLAST tool (https://blast.ncbi.nlm.nih.gov/Blast.cgi). The pathogenicity of the identified variants was then assessed according to “Standards and Guidelines for the Interpretation of Sequence Variants, Version 2015,” issued by the American Association of Medical Genetics and Genomics (Richards et al., 2015).

### 2.5 Prenatal diagnosis and biochemical analysis

The fetus of the proband was monitored by ultrasonography throughout pregnancy. The thickness of nuchal translucency (NT) was 0.37 cm at 11^+4d^ gestational weeks (Figure 2h), but no other specific intrauterine phenotype was evident. Amniocentesis was performed at 17^+1d^ gestational weeks, followed by a combination of genetic detection, including chromosomal karyotyping, chromosome microarray analysis (CMA; Yang, Shen, et al., 2019), and Sanger sequencing.

Subsequent to the receipt of a positive genetic result with respect to the fetus at 21 weeks, the couple decided to terminate the pregnancy at 21^+3d^ weeks. The appearance of the fetus had no obvious contracture and other abnormalities. Subsequently, we obtained muscle gastrocnemius tissue from the fetus and performed western blotting (WB, with anti‐heavy chain myosin/*MYH3* antibody, ab124205) along with a control sample from a normal fetus selectively aborted at ~20 gestational weeks, and image data were processed as described in our previous study (Yang et al., 2020). Meanwhile, pathological staining with hematoxylin and eosin (HE), Gomori trichrome (GT), and succinate dehydrogenase (SDH) on it were conducted in accordance with the respective manufacturers’ protocols.

## 3 RESULTS

### 3.1 WES and variant analysis

**Figure 3:**
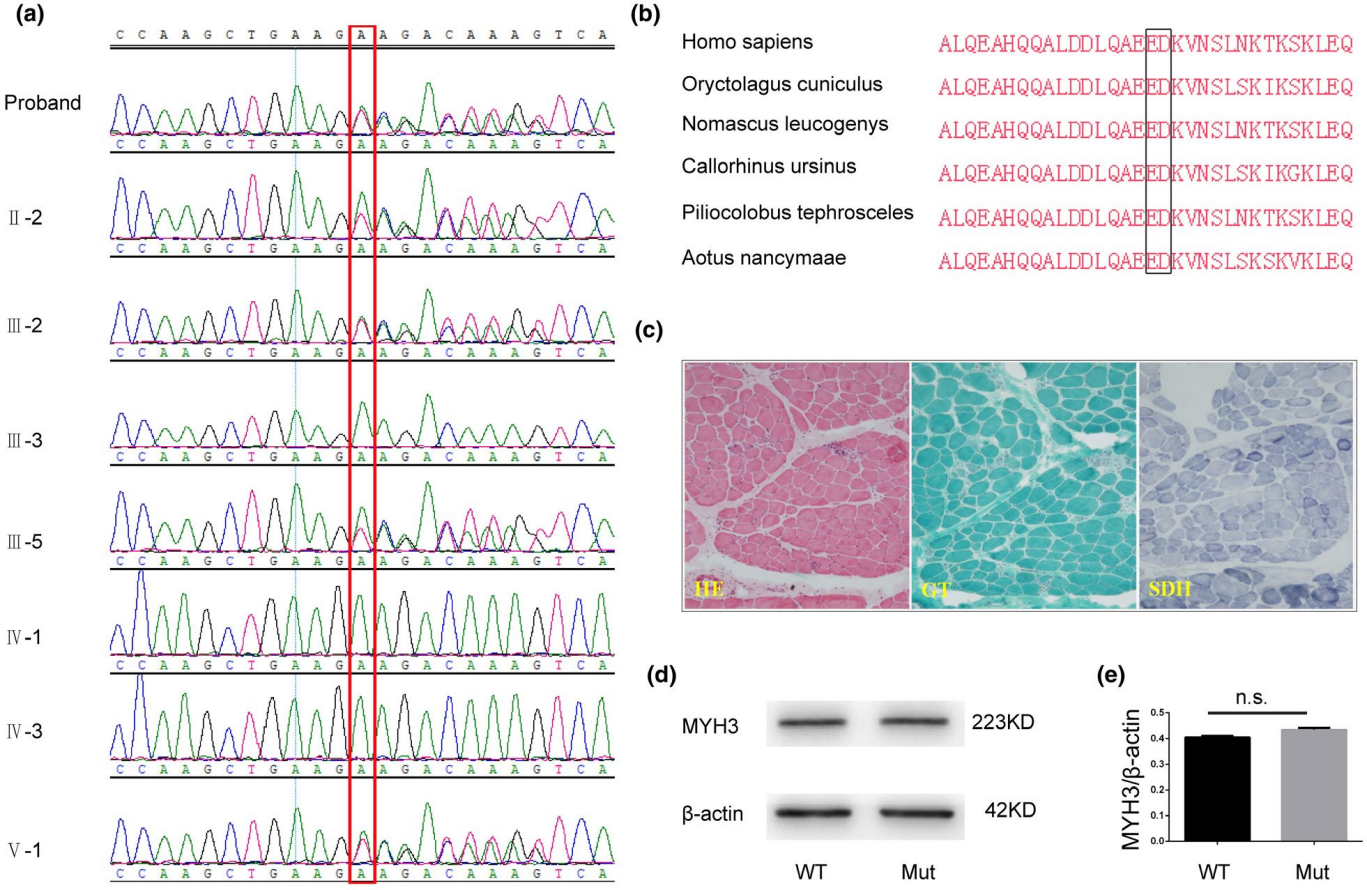
Results of genetic and biochemical tests: (a) Sanger sequencing chromatograms indicating the variant c.3044_3047delinsTCAATTTGTT (references NG_011537.1). (b) Conservation of amino acids *MYH3*: p.E1015_D1016 across species from NABI‐BLAST. (c) Result of pathological staining by hematoxylin and eosin (HE), Gomori trichrome (GT), and succinate dehydrogenase (SDH). (d) WB result of *MYH3* expression in skeletal muscle samples of the control (WT) and the affected fetus (Mut). (e) Quantitative analysis of *MYH3*/beta‐actin between the control (WT) and the affected fetus (Mut) showed no significant difference (*p* > 0.05)

WES analysis revealed a novel heterozygous pathogenic variant NM_002470.3: exon24: c.3044_3047delinsTCAATTTGTT: p.E1015_D1016delinsVNLF in the *MYH3* gene harbored by the proband (Ⅳ:2) and four other members (Ⅱ:2; Ⅲ:2; Ⅲ:5; and Ⅴ:1 [fetus]) but not by the other three unaffected members (Ⅲ:3; Ⅳ:1; and Ⅳ:3). This was confirmed by Sanger sequencing (Figure 3a). The BLAST result demonstrated that the amino acids p.E1015_D1016 remained highly conserved among species (Figure 3b). According to the ACMG guidelines, this variant was interpreted as pathogenic on evidence of PM2+PM4+PP1+PP3+PP4 (Richards et al., 2015). Except with this variant, the fetal chromosomal karyotype and CMA test results were normal.

### 3.2 WB and pathological staining

Pathological staining results with all three dyes showed no obvious change in fetal skeletal muscle fibers (Figure 3c; Cuisset et al., 2013). Additionally, the WB result indicated that there was no significant difference in *MYH3* expression in the sample from the affected fetus as compared to the control (Figure 3d,e).

## 4 DISCUSSION

The myosin superfamily is a highly conserved versatile group of molecular motors involved in the transport of specific biomolecules, vesicles, and organelles in eukaryotic cells (Syamaladevi, Spudich, & Sowdhamini, 2012). Along with an actin filament, its transport function is achieved by generating physical force from the chemical energy of ATP hydrolysis (Pokrzywa et al., 2015; Syamaladevi et al., 2012). The myosin involved in myofilaments in rhabdomyocytes, smooth muscle, and non‐muscle cells is myosin class II, whose heavy chain (MyHC) is encoded by the *MYH* gene family (Sellers, 2000). The *MYH3* gene encodes an embryonic isoform (MyHC‐emb) expressed in the fetal musculature, which plays an important role in the formation of normal vertebrae and in the regulation of fetal muscle contraction and relaxation.11 Moreover, it can be mediated by actin‐binding protein and connected with the cell membrane to maintain the stability of cytoskeleton (Tajsharghi & Oldfors, 2013). Two MyHC isoforms are expressed during fetal development in humans: embryonic MyHC, as encoded by *MYH3*; and perinatal MyHC, as encoded by *MYH8* (MIM *160741; Schiaffino & Reggiani, 2011).

**Figure 4:**
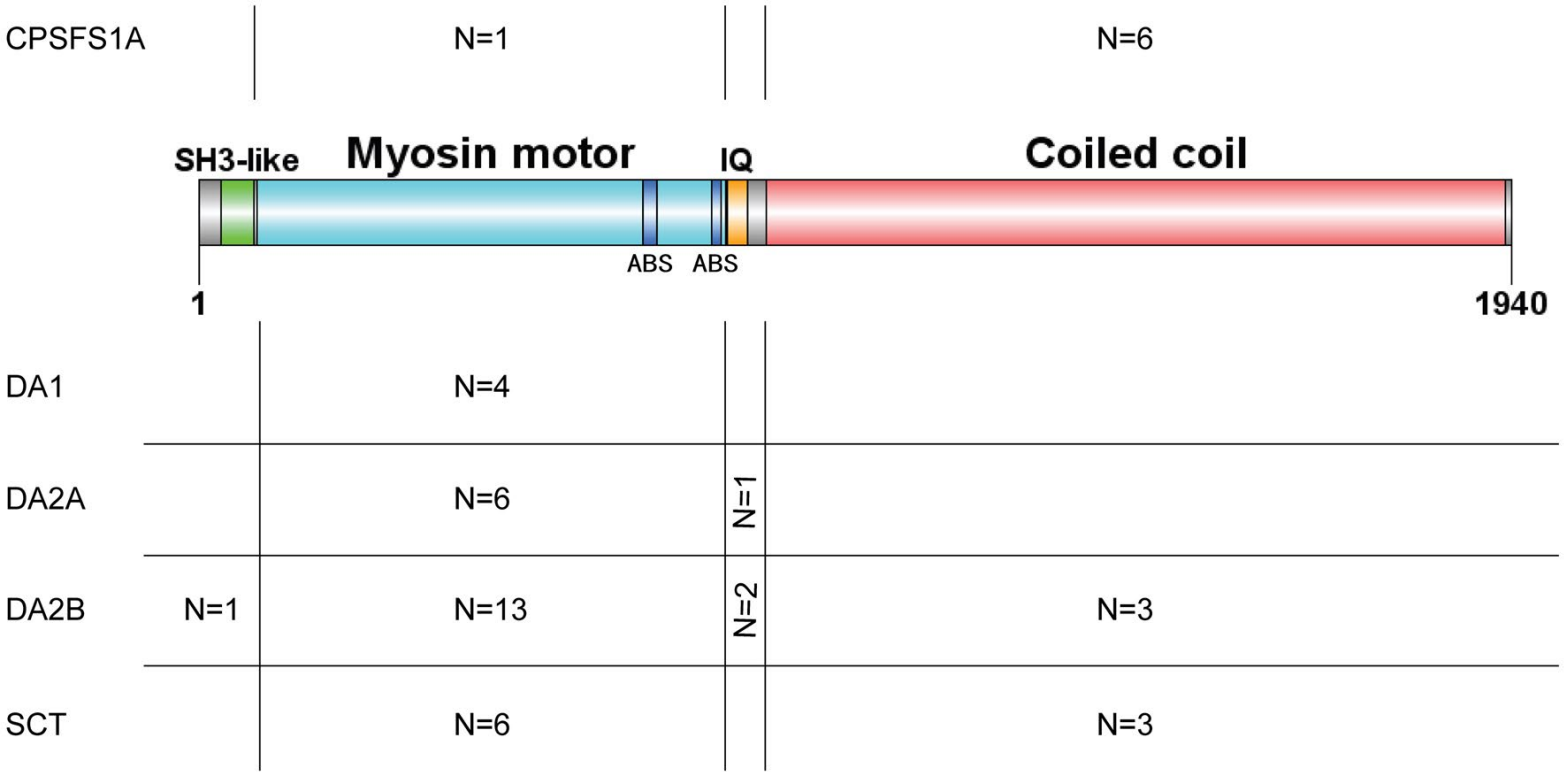
Distribution count for pathogenic variants of various DAs in specific domains of the *MYH3* peptide chain. ABT, actin‐binding site. DA, distal arthrogryposis. CPSFS1A, contractures, pterygia, and spondylocarpotarsal fusion syndrome 1A. SCT, spondylocarpotarsal synostosis syndrome. (Note: There are four variants that are not in the coding region and are not shown in this figure. See details in Table S1.)

Human *MYH3* is located in cytogenetic location 17p13.1, contains 41 exons, and encodes a 1940 aa peptide chain (XP_011522172.1 in NCBI blast). MyHC can be divided into two major domains: the globular amino‐terminal head, which is responsible for binding to the myosin light chain and to actin as well as for ATP hydrolysis; and the alpha helical carboxy‐terminal rod, which is responsible for the ability of myosin to form filaments (Tajsharghi & Oldfors, 2013). The novel variant identified in this study is located in the coiled‐coil domain in the tail, a type of secondary structure composed of two or more alpha helices, which entwines to form a cable structure and serves a mechanical role in forming stiff bundles of fibers (Alva, Syamala Devi, & Sowdhamini, 2008). In previous studies, 50 pathogenic variants in the *MYH3* gene associated with various DA disorders have been reported, which were distributed in various structural domains of MyHC‐emb as shown in Figure 4 and Table S1 (Bae et al., 2016; Beck et al., 2013; Fu et al., 2018; Hague et al., 2016; Monaghan et al., 2020; Tajsharghi et al., 2008; Todd et al., 2015; Toydemir et al., 2006; Wang, Kong, Zuo, & Kang, 2020; Xu, Kang, & Zhang, 2018). The clinical classification of DA disorders associated with *MYH3* variants or the genotype‐phenotype correlations, as documented in numerous reports, was unclear and therefore required additional clarification. Overall, however, there are some differences between DA1/2's pathogenic variants and CPSFS1A's distribution within specific domains of *MYH3*, which may explain the phenotypic differences in these disorders (Figure 4).

Few reports have described the CPSFS1A (Carapito et al., 2016; Chong et al., 2015; Scala et al., 2018) and CPSFS1B (Cameron‐Christie et al., 2018) conditions caused by *MYH3*. However, in this study, we detected a novel heterozygous variant c.3044_3047delinsTCAATTTGTT in *MYH3* in a five‐generation Chinese family presenting typical features of CPSFS1A. This variant resulted in a change in protein length (but not in reading frames), and the relevant amino acids E1015_D1016 were evolutionarily conservative. This variant meets the evidence level of PM2 (0 frequency in the databases of 1000G (https://www.internationalgenome.org/), gnomAD (http://gnomad.broadinstitute.org/), and Berry Genomics in‐house database), PM4 (resulting in a change in the length of the protein but not in the reading frame), PP1 (cosegregation of variant and phenotype), PP3 (evolutionary conservatism, potential damage to protein), and PP4 (the disease caused by the mutated gene matches the phenotype of this case), so it could be interpreted as “Pathogenic.” Presumably, the phenotypic variability of *MYH3* related DAs is probably attributable to different polymorphism backgrounds between patients or functional differences in molecules interacting with MyHC‐emb (Cameron‐Christie et al., 2018; Yang, Wu, et al., 2019) In any case, the mechanism behind CPSFS1 needs further delineation in larger groups of study subjects.

The fact that the affected fetus did not show a severe phenotype like any other patient's at the twenty‐first gestational week seemed to coincide with the results of biochemical experimentation, suggesting that the manifestations of CPSFS1 might be aggravated dynamically during late pregnancy or perinatal period. However, this inference should be supported by more solid evidence, such as the results of protein function studies.

WES provides an accurate method in the prenatal diagnosis of CPSFS1A. The couple was counseled on management options including chromosomal karyotype、CMA and WES. Prenatal diagnosis indicated that the fetus in this family carried the same variant as the proband, so the fetus was presumably to be a patient. The parents decided to terminate the pregnancy. Following the termination of the pregnancy, the result of gene analysis of the aborted tissues was consistent with prenatal diagnosis. As can be seen in numerous diagnostic studies (Fu et al., 2018; Monaghan et al., 2020; Wang & Yuan, 2017; Yang, Shen, et al., 2019), on the bases of methodology and time consumption, WES is an effective method for rapid acquisition of pathogenic variants. For any future pregnancy of the proband in this study, the recurrent risk of CPSFS1 condition would be 50%. Given such circumstances, the couple was informed of reproductive options such as prenatal testing and preimplantation genetic diagnosis (PGD).

In conclusion, our study has first detected a novel heterozygous variant c.3044_3047delinsTCAATTTGTT in *MYH3* in a five‐generation Chinese family presenting typical features of CPSFS1A. Our results expand the variant spectrum of *MYH3* CRYGC mutations, which may further be helpful in the molecular diagnosis of CPSFS1A. Additionally, we avoided the birth of the affected fetus by prenatal diagnosis. Additional in‐depth research is necessary in order to investigate the pathogenesis of CPSFS1A.

## CONFLICT OF INTERESTS

The authors declare that they have no competing interests.

## AUTHOR CONTRIBUTIONS

Jing Zhang: Project administration, Conceptualization, Methodology. Wen‐Qi Chen: Writing‐ Original draft preparation. Si‐Wen Wang: Validation, Investigation. Shao‐Xiong Wang: Data Curation, Visualization. Mei Yu: Resources. Qing Guo: Supervision. Ya‐Dong Yu: Writing‐ Reviewing and Editing.

## Supporting information

Table S1

## Data Availability

All available clinical data are shared in the article.
