# Supplementary material for: Identification of a novel pathogenic variant in the MYH3 gene in a five‐generation family with CPSFS1A (Contractures, Pterygia, and Spondylocarpotarsal Fusion Syndrome 1A)
Source: Mol Genet Genomic Med. 2020 Aug 7;8(10):e1440. doi: 10.1002/mgg3.1440 (PMC7549579; doi:10.1002/mgg3.1440)
Supplement: Supplementary file 1 — Table S1 [file MGG3-8-e1440-s001.docx]

**Supplementary Table 1 *MYH3* variants in various DA disorders**

| **Disorder** | **Nucleotide change** | **Exon** | **Protein change** | **Domain** | **References** |
| --- | --- | --- | --- | --- | --- |
| **DA1** | 551G>C | 7 | G184A | Myosin motor | pmid:23401156 |
|  | 700G>A | 8 | A234T | Myosin motor | pmid:23401156 |
|  | 1309T>A | 14 | F437I | Myosin motor | pmid:21531865 |
|  | 1512G>T | 15 | K504N | Myosin motor | pmid:23401156 |
| **DA2A** | 602C>T | 6 | T178I | Myosin motor | pmid:16642020;  30826400 |
|  | 1160A>G | 13 | T387C | Myosin motor | pmid:25256237 |
|  | 1562A>G | 15 | E498G | Myosin motor | pmid:16642020 |
|  | 1817A>C | 16 | Y583S | Myosin motor | pmid:16642020 |
|  | 2083C>T | 18 | R672C | Myosin motor | pmid:16642020 |
|  | 2084G>A | 18 | R672H | Myosin motor | pmid:16642020 |
|  | 2543T>A | 22 | V825D | IQ | pmid:16642020 |
| **DA2B** | 118G>A | 3 | V40M | SH3-like | pmid:31030430 |
|  | 602C>T | 6 | T178I | Myosin motor | pmid:16642020 |
|  | 700G>A | 8 | A234T | Myosin motor | pmid:18695058;  23401156 |
|  | 737G>C | 9 | G246A | Myosin motor | pmid:23401156 |
|  | 851C>T | 9 | S261F | Myosin motor | pmid:16642020 |
|  | 859T>G | 10 | P287V | Myosin motor | pmid:29314551 |
|  | 944C>G | 10 | S292C | Myosin motor | pmid:16642020;  pmid:28779239 |
|  | 1019T>A | 12 | L340Q | Myosin motor | pmid:23401156 |
|  | 1192G>A | 12 | E375K | Myosin motor | pmid:16642020 |
|  | 1160A>G | 13 | T387C | Myosin motor | pmid:29625835 |
|  | 1385A>G | 14 | D462G | Myosin motor | pmid:18695058 |
|  | 1397T>G | 14 | F466C | Myosin motor | pmid:23401156 |
|  | 1618G>T | 15 | D517Y | Myosin motor | pmid:16642020 |
|  | 2375G>T | 21 | G769V | Myosin motor | pmid:16642020 |
|  | 2503_2505delTTC | 22 | F835del | IQ | pmid:2340115 |
|  | 2581A>G | 22 | K838E | IQ | pmid:16642020 |
|  | 2590_2592delCTC | 22 | L841del | Coiled coil | pmid:16642020 |
|  | 4934A>C | 34 | D1622A | Coiled coil | pmid:16642020 |
|  | 4979C>T | 34 | A1637V | Coiled coil | pmid:16642020 |
| **CPSFS1A** | 727_729del | 8 | S243del | Myosin motor | pmid:25957469 |
|  | 3044_3047delinsTCAATTTGTT | 24 | E1015_D1016delinsVNLF | Coiled coil | novel |
|  | 3214_3216dupAAT | 25 | N1072dup | Coiled coil | pmid:25957469 |
|  | 3224A>C | 25 | Q1075P | Coiled coil | pmid:25957469 |
|  | 4031T>C | 30 | L1344P | Coiled coil | pmid:27381093 |
|  | 5263_5265delCCA | 36 | Lys1755del | Coiled coil | pmid:30008475 |
|  | 5355_5357delGAA | 37 | Lys1785del | Coiled coil | pmid:30008475 |
| **SCT** | -9+1G>A | - | - | 5'-UTR |  |
|  | 141T>G | 3 | p.Y47* | Myosin motor | pmid:29805041 |
|  | 721_725delAACTCinsGACAA | 8 | N241D:S242N | Myosin motor | pmid:29805041 |
|  | 727_729delTCC | 8 | S243del | Myosin motor | pmid:28205584 |
|  | 998C>G | 11 | T333R | Myosin motor | pmid:27381093 |
|  | deletion of intron12 and exon25 | - | - | / | pmid:29805041 |
|  | 1581+1G>A | - | - | / | pmid:29805041 |
|  | 1934T>G | 17 | F645C | Myosin motor | pmid:28205584 |
|  | 1986_1990delTTTAA | 18 | N662Kfs*15 | Myosin motor | pmid:29805041 |
|  | 2699delT | 23 | L900fs*9 | Coiled coil | pmid:28205584 |
|  | 4031T>C | 30 | L1344P | Coiled coil | pmid:27381093 |
|  | 4647+1G>A | - | - | / | pmid:29805041 |
|  | 5198_5205dupCAGACCTC | 36 | M1736Qfs*10 | Coiled coil | pmid:30228365 |
